# Strategic threat health response in Australia capital cities: Predicting the optimum locations for field hospitals through geospatial analysis

**DOI:** 10.1002/puh2.177

**Published:** 2024-05-08

**Authors:** Mazen Baazeem, Estie Kruger, Marc Tennant

**Affiliations:** ^1^ International Research Collaborative – Health and Equity, School of Allied Health The University of Western Australia Crawley Western Australia Australia; ^2^ Ministry of Health Makkah Health Cluster– Maternity and Children's Hospital Makkah Saudi Arabia

**Keywords:** COVID‐19, field hospitals and healthcare accessibility, GIS, health policy, public health preparedness

## Abstract

**Objective:**

The advent of the COVID‐19 pandemic has accentuated the critical importance of epidemic preparedness within national healthcare systems. This study presents a geospatial analysis aimed at optimising the placement of field hospitals in Australia to ensure adequate healthcare access during pandemics.

**Methods:**

The latest census data from the Australian Bureau of Statistics were integrated with the spatial locations of current emergency departments within Australian capitals. Buffer zones with a 7.5 km radius were created around the public emergency departments (EDs). Buffer zones outside the 7.5 km radius that exhibited high population densities were categorised into high‐density (red), moderate‐density (yellow) and low‐density (green) zones to prioritise and tailor healthcare responses during a pandemic. The identification of high‐density population areas outside the ED radius enabled the stratification of potential sites for ED facilities.

**Results:**

Ninety‐one potential field hospital sites were identified across Australia's capital cities. The findings indicate that the addition of these sites would place over 95% of the population within a 7.5 km radius of an ED facility. This network of proposed sites is designed to serve a spectrum of population densities, ensuring equitable healthcare access for both densely populated urban centres and less populated areas.

**Conclusions:**

This study underscores the potential of field hospitals to strengthen Australia's public health system against emergencies. By advocating for the creation of 91 field hospitals within a 7.5 km reach for over 95% of urban dwellers across major cities, it demonstrates a strategic approach to ensure comprehensive ED coverage. Drawing on international examples, including China's Fangcang hospitals, the USA's post‐acute care (PAC) facilities and the United Kingdom's National Health Service (NHS) Nightingale Hospitals, it highlights the need for healthcare agility and scalability, especially during pandemic outbreaks. The research presents a blueprint for field hospital deployment, marking a significant advancement in public health logistics and protection across Australia's varied demographic and geographical landscapes.

## Graphical abstract

The strategic placement of field hospitals in Australia's capital cities has been carefully planned to ensure maximum coverage in potential emergencies, aiming to provide health services to 95% of the population. This critical positioning is designed to meet healthcare demands during a pandemic, offering swift medical access to the majority of the urban population (graphical illustration image created with AI).

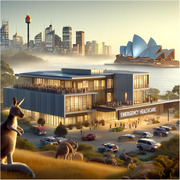

## INTRODUCTION

As the frequency and impact of global health threats like pandemics escalate, the imperative for preparedness becomes increasingly pronounced. The last few decades have been punctuated by a series of infectious disease outbreaks that have spanned continents and strained healthcare systems, the most recent and pervasive being the COVID‐19 pandemic, caused by the severe acute respiratory syndrome coronavirus 2 [1]. In the face of such large‐scale health crises, Australia's geographic vastness and the contrasting densities of its population distribution pose distinct challenges to the timely and effective delivery of healthcare services [2, 3, 4]. Despite the lessons gleaned from past outbreaks, such as H1N1 influenza in 2009 and the subsequent global epidemics of SARS and Middle East respiratory syndrome (MERS), the COVID‐19 pandemic exposed significant gaps in the nation's healthcare preparedness and infrastructure, necessitating an urgent reassessment of strategic response mechanisms [5, 6, 7].

The pandemic's pressure on healthcare systems was acutely felt in Australia, where the rapid case escalation led to the overcrowding of emergency departments and intensive care units (ICUs), particularly in heavily impacted regions like Victoria [5]. This situation was exacerbated by global supply chain vulnerabilities, which precipitated a shortage of critical resources such as personal protective equipment (PPE), amplifying the risks for healthcare professionals [8]. The exigencies of the pandemic compelled the deferral of elective surgeries and non‐urgent medical procedures, engendering extended waiting periods for patients and raising concerns over the potential neglect of other severe health conditions, including delayed cancer diagnoses [9, 10]. Amidst these challenges, a notable shift occurred with the accelerated adoption of telehealth services, an initiative expanded by the Australian government to mitigate virus transmission risks and maintain continuity of care [11].

In response to the heightened demand for healthcare services and the need for rapid, adaptive solutions during pandemics, field hospitals and health camps emerged as critical components of the medical response. These temporary healthcare settings, serving as field hospitals, provided an immediate resolution to the prevalent issue of bed shortages during outbreaks. With their modular and scalable design, field hospitals acted as a critical buffer in healthcare provisioning, enabling the expansion or contraction of services in accordance with the evolving needs of affected populations. Furthermore, these installations played a crucial role in delineating patient populations, thereby reducing the risk of cross‐infection and optimising the deployment of specialised resources [12]. The global experiences during the COVID‐19 pandemic, particularly China's implementation of Fangcang shelter hospitals, in the United States Boston's post‐acute care (PAC) hospital and the United Kingdom's establishment of National Health Service (NHS) Nightingale Hospitals, offer insightful precedents for the role of rapid, scalable and adaptable healthcare responses in crisis situations [13, 14, 15]. These interventions provided additional healthcare capacity and underscored the critical nature of flexibility and swift action in public health emergency management.

This research seeks to investigate the strategic placement of field hospitals to bolster the epidemic preparedness of the Australian public health system. By analysing current infrastructure, demographics data and potential threats, it will delineate recommendations for the optimal positioning of future hospital facilities to counteract emergent public health crises effectively.

## METHODS

This analysis was conducted across multiple greater capital city regions within Australia, encompassing a diverse range of environments, including urban, suburban and semi‐rural areas. The setting was selected based on its unique demographic and geographic characteristics, representing a broad spectrum of the Australian population. This diverse selection ensures the findings are applicable to various contexts, both within Australia and potentially in similar settings internationally.

### Data acquisition and preparation: population

This study used the most recent census data available from 2021, explicitly employing Statistical Area Level 1 (SA1) datasets, which were obtained from the Australian Bureau of Statistics (ABS). These datasets covered Australian States and Territories, including New South Wales, South Australia, Victoria, Queensland, Tasmania, Western Australia, the Australian Capital Territory and the Northern Territory. SA1s are the smallest unit for which census data are released, typically encompassing an average population of approximately 400 individuals. This granularity allowed for a detailed analysis of population distribution across the regions. To enhance the focus and applicability of the study, this analysis was confined to the greater metropolitan areas within the Australian States and Territories. This delimitation was crucial in shaping our assessment of regions with higher population densities and more complex healthcare infrastructure needs, which are typically characteristic of metropolitan landscapes.

### Data acquisition and preparation: existing health infrastructure

In conjunction with population data, the spatial locations of public hospitals equipped with emergency departments within the nation's capital cities were sourced from the Department of Health and Aged Care website [16].

### Geospatial analysis

The acquired SA1 population data and public hospital locations were integrated into the Quantum Geographic Information System (QGIS version 3.24) for advanced spatial mapping and analytical processes. The integration of these datasets facilitated a comprehensive assessment of the spatial distribution of healthcare facilities relative to population arrangement.

### Determining accessible distance

For the purposes of this study, a 7.5 km spatial radius was established around each public emergency department (ED) facility. This distance was identified as the most accessible for both public transport and motorists in emergency scenarios, based on findings from previously published studies [3, 17, 18].

### Population density stratification

To address areas falling outside the initial 7.5 km radius, secondary buffer zones were established to capture areas with high population densities. These buffers were applied to identify and prioritise regions that, despite their distance from established emergency departments, exhibited substantial population figures and, therefore, represented critical areas of need for additional healthcare services during a pandemic.

### Visualisation and site prioritisation

The prioritisation of potential field hospital deployment locations was visualised using a colour‐coding scheme based on the population density within the secondary buffer zones. The most densely populated areas were marked in red, indicating the highest priority. Zones with moderate population densities were illustrated in yellow, and those with the least density were shown in green. This visual stratification system allowed for an immediate, clear indication of where the deployment of field hospitals would be most beneficial in mitigating healthcare service gaps during a pandemic.

## RESULTS

The strategic deployment of field tent hospitals across Australia's greater capital city regions was meticulously mapped out to ensure maximal coverage during a potential national or capital city emergency scenario. The establishment of 91 such facilities would ensure that over 95% of the population within these urban locales would be within a 7.5 km radius of an ED. This positioning is critical to meet the healthcare demands that could arise during a pandemic, providing expedient access to medical care for the majority of the urban populace (Table 1).

**TABLE 1:** Number of populations covered with the current situation and after the proposed field hospitals.

	Field hospitals			Public ED coverage		Field hospital coverage		Total covered population		2021 population
City	Red	Yellow	Green							
Canberra	2	1	0	167,295	37%	260,134	57%	427,429	94%	454,499
Sydney	11	2	2	3831,756	73%	1187,866	23%	5019,622	96%	5231,147
Melbourne	12	4	4	2845,434	58%	1654,589	34%	4500,023	92%	4917,750
Brisbane	17	6	2	930,312	37%	1470,083	58%	2400,395	95%	2526,238
Hobart	0	1	5	75,076	30%	140,745	57%	215,821	87%	247,086
Adelaide	4	2	0	873,360	63%	412,027	30%	1285,387	93%	1387,290
Darwin	2	0	0	27,083	19%	95,767	68%	122,850	88%	139,902
Perth	5	6	3	836,127	40%	1156,103	55%	1992,230	94%	2116,647
Total	53	22	16	9586,443	56%	6377,314	37%	15,963,757	95%	17,020,559

Abbreviation: ED, emergency departments.

In Perth, 14 strategically positioned field hospital sites have been identified to serve a population of 1156,103 (Table 1). The spatial delineation revealed five high‐density areas, colour‐coded red, and six moderate‐density areas, colour‐coded yellow. This differentiation allows for a tiered healthcare response that aligns resources with population density (Figure 2).

The study recognises the necessity for a robust infrastructure to support field hospitals, especially during pandemics or emergencies. Nominal locations were selected based on their existing amenities, such as space availability, accessibility and the potential for upgrading essential services like electricity and sewerage systems. For instance, using the Perth metropolitan area as an example, a centrally located oval in one of the red zones, already equipped with basic infrastructure, was identified as an ideal candidate for rapid conversion into a field hospital (Table 2). The feasibility of enhancing its existing facilities to meet healthcare standards was evaluated, considering factors like power supply reliability, water and sewage system capacity and ease of access for emergency vehicles and the public. This methodical selection of nominal locations ensures that, in the event of a health emergency, the transition to operational field hospitals can be swift and efficient, thereby significantly reducing the lead time for emergency healthcare provisioning in Perth's most densely populated areas.

**TABLE 2:** The suggested nominal locations for field hospitals in the greater Perth metropolitan area.

Green zones		
Nominal locations	latitude	longitude
Harry Riseborough Oval	−31.90149338	116.1730954
Kostera Reserve	−31.97621355	116.0555426
Briggs Park	−32.22618707	116.0019998

**Yellow zones**		
Nominal locations	Latitude	longitude
Coolamon Oval	−31.77687957	115.9759487
Beale Park	−32.10026753	115.7777096
McWhirter Oval	−32.2678675	115.8466353
Barri Barri Reserve	−32.34038554	115.8210014
Rhonda Scarrott Oval	−32.42262985	115.7571653
Falcon Reserve	−32.57937352	115.6617438

**Red zones**		
Nominal locations	Latitude	longitude
Fred Marsh Oval	−31.8538291	115.7761756
Kingfisher Oval	−31.83709259	115.8937753
Quinns Districts Amateur Football Club	−31.66944503	115.7246247
Thornlie Oval	−32.05792564	115.9550557
Harvest Lakes Community Centre	−32.15166662	115.8650801

**FIGURE 1:**
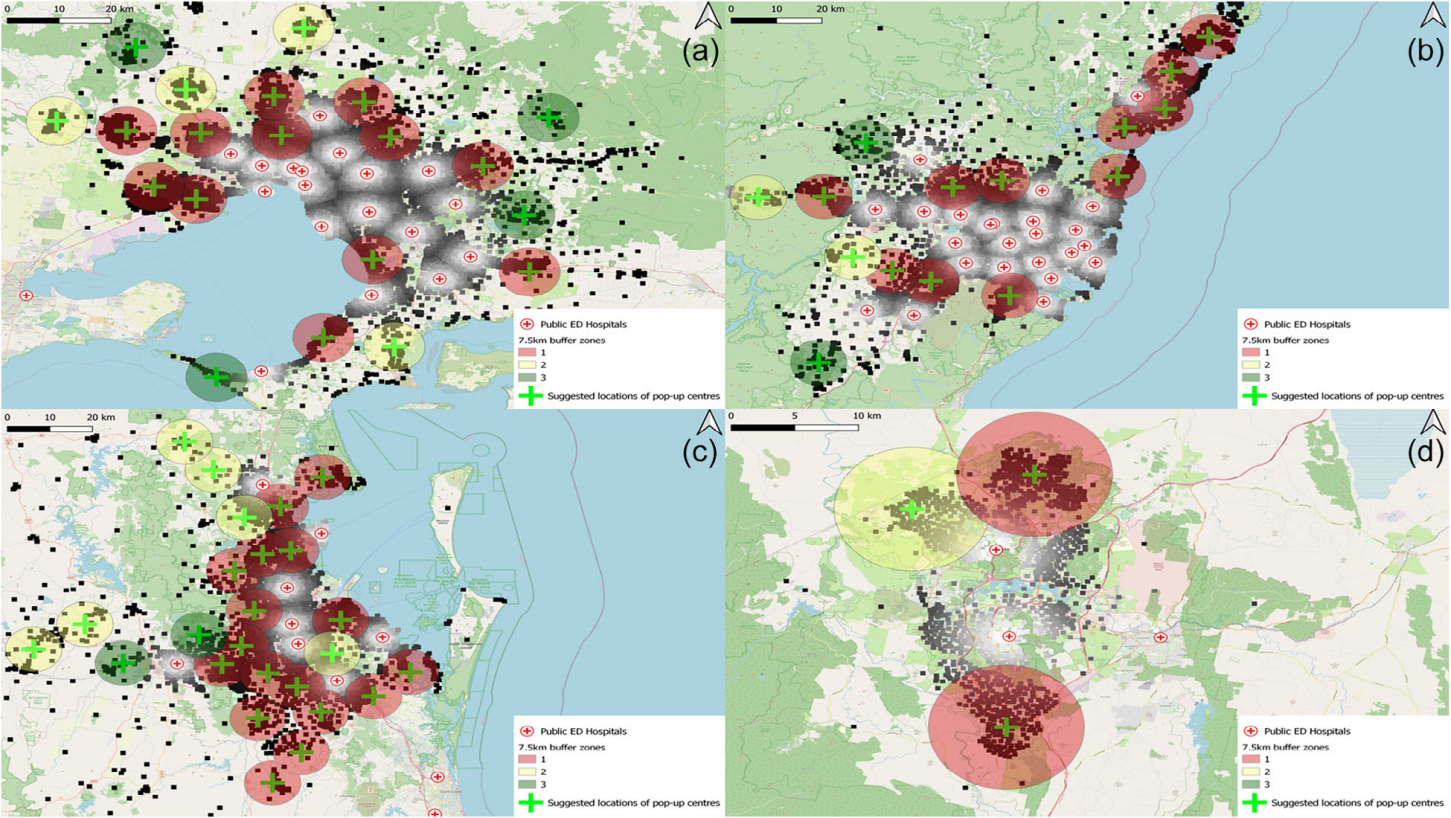
Suggested field hospitals with 7.5 km buffer zones in high population density areas of (a) Melbourne, (b) Sydney, (c) Brisbane and (d) Canberra, categorised into high‐density (red), moderate‐density (yellow) and low‐density (green) zones.

Sydney, with its sprawling urban expanse and the highest population among Australian cities, would have 15 sites, effectively covering 1187,866 residents (Table 1). The data presented 11 high‐density red zones, underscoring the city's significant need for accessible healthcare facilities in a pandemic. Additionally, two yellow zones of moderate density were identified, ensuring comprehensive coverage (Figure 1).

Melbourne's plan incorporates 20 sites to cater to its 1654,589 inhabitants (Table 1). The analysis identified 12 red zones, indicating a high concentration of the population and four yellow zones. This distribution is reflective of Melbourne's urban density and the necessity for an extensive healthcare response framework (Figure 1).

**FIGURE 2:**
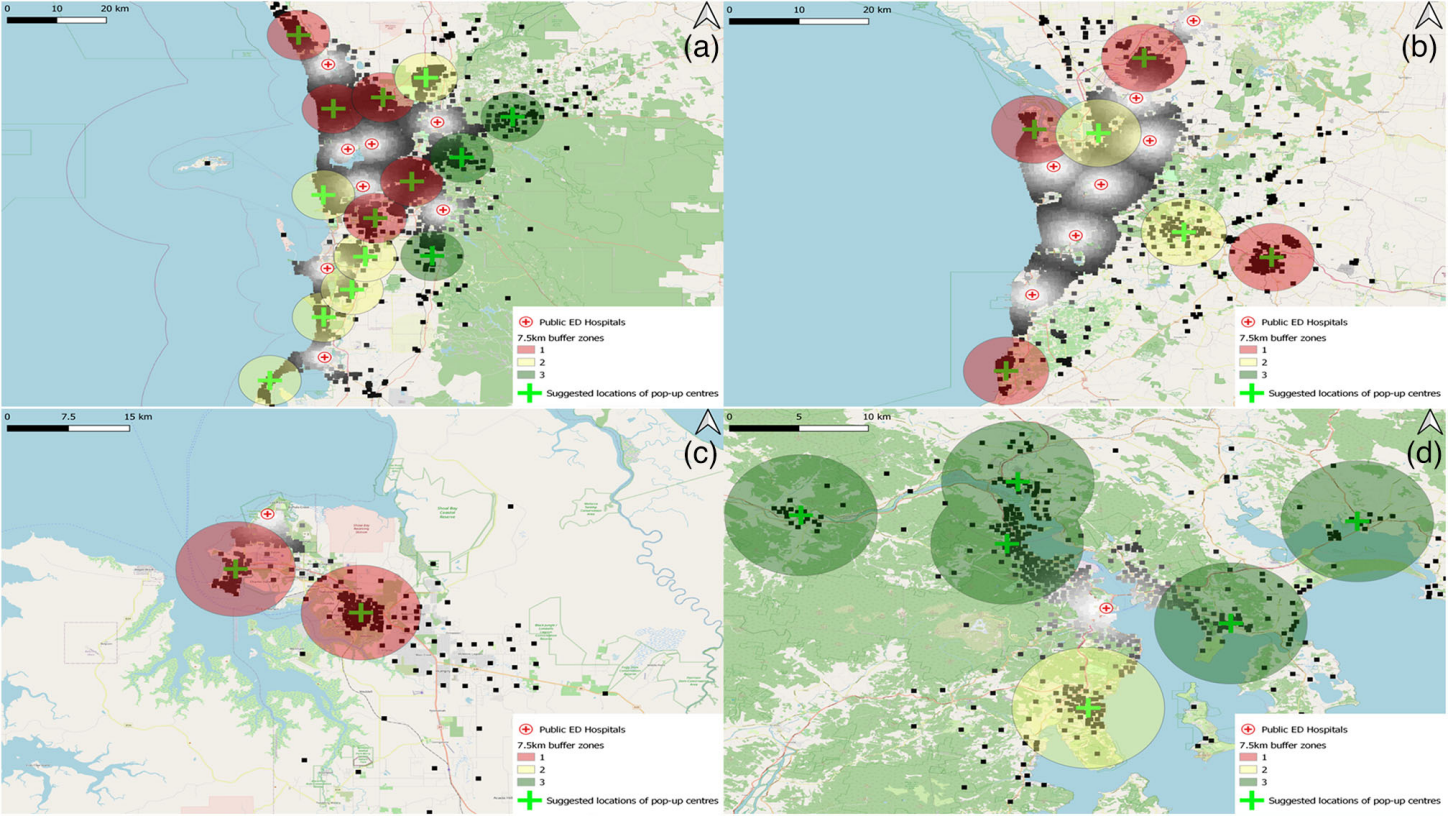
Suggested field hospitals with 7.5 km buffer zones in high population density areas of (a) Perth, (b) Adelaide, (c) Darwin and (d) Hobart, categorised into high‐density (red), moderate‐density (yellow) and low‐density (green) zones.

In Hobart, 6 sites are designated for 140,745 individuals, ensuring comprehensive reach within the smaller capital (Table 1). A lower overall population density resulted in five green zones, indicative of lower population density, and one yellow zone, suggesting a moderate population density. This finding aligns with Hobart's smaller urban footprint and lower healthcare facility demand during a pandemic (Figure 2).

The analysis revealed two high‐density red zones, indicative of Darwin's central urban areas, where field hospitals would be most critical in providing immediate care during a healthcare surge (Figure 2). Establishing two sites will relatively cover the population of 95,767 (Table 1).

Canberra's plan includes 3 sites to serve 260,134 people (Table 1). The distribution encompassed two high‐density red zones and one moderate‐density yellow zone, reflecting the city's planned urban structure and the corresponding healthcare service requirements (Figure 1).

Brisbane's larger population requires a more extensive network of 25 field hospitals. The prevalence of red zones necessitates a particularly robust pandemic healthcare provisioning plan for Brisbane. The mapping exercise pinpointed 17 red zones, the highest number of high‐density areas among all analysed cities, and six yellow zones (Figure 1). It will provide coverage for 1470,083 individuals (Table 1).

Lastly, Adelaide's coverage is secured with 6 sites, accommodating a population of 412,027 with 4 high‐density red zones and 2 moderate‐density yellow zones, delineating the areas within the city that would most likely necessitate rapid healthcare scaling in response to an emergency (Table 1) (Figure 2).

## DISCUSSION

Through the integration of detailed population data and public emergency healthcare locations within QGIS, this study established a methodology for assessing and prioritising potential sites for field hospitals. The approach ensured that both population density and proximity to existing healthcare facilities were considered, providing a nuanced framework for emergency healthcare planning in the context of Australian States and Territories. The analysis reveals a targeted healthcare provisioning approach that is equitable and efficient. High‐density zones, identified as red, represent areas of critical need that would benefit most from the immediate availability of field hospitals. Moderate‐density, identified as yellow‐coded zones, and low‐density, identified as green‐coded areas, would also be supported, albeit with a scaled approach that aligns resources with anticipated demand. This stratification is pivotal for operational readiness and resource optimisation during a health crisis. The allocation of these field hospitals within the stipulated buffer zones underscores a commitment to ensuring healthcare accessibility in the face of a pandemic. This approach aligns with proactive public health planning, aiming to mitigate the risk of overwhelming the existing healthcare infrastructure during times of crisis.

In an era where global health threats like pandemics are becoming increasingly prevalent, preparedness is crucial. Australia, with its vast geography, characterised by densely populated coastal areas and expansive rural regions, poses unique challenges in healthcare provisioning during epidemics [19]. This geographic reality and the potential for rapidly spreading infections require a dynamic response. Hospitals in regions that witnessed substantial outbreaks, like Victoria, experienced acute pressure from the pandemic [9]. The significant surge in cases led to overcrowding in emergency departments and ICUs, highlighting potential limitations in the system's capacity to accommodate an unexpected influx [5]. Concurrently, the pandemic underscored vulnerabilities in patients awaiting non‐emergency interventions and faced prolonged waiting times, with some experiencing deteriorating health conditions. This omnipresent fear of the virus results in a segment of the population avoiding medical facilities.

Amidst this backdrop, adaptive healthcare solutions, notably field hospitals and health camps or tents, have emerged as critical factors in ensuring optimal medical response during pandemics. Field hospitals offer an immediate remedy to the issue of hospital bed shortages, a common predicament during widespread outbreaks. Despite their comprehensive resources, established healthcare facilities can quickly reach or exceed their capacity when faced with an unprecedented surge in patients. The rapid deployment of hospitals can serve as a buffer, accommodating patients who might otherwise be left without immediate care. Their modular and scalable nature ensures that they can be expanded or contracted based on the immediate needs of the affected region. Moreover, these makeshift medical establishments play a pivotal role in segmenting patient populations. The risk of cross‐infection within hospitals can be minimised by designating certain facilities exclusively for pandemic‐affected patients. This not only protects patients with other medical conditions but also allows for a more focused allocation of specialised resources and personnel to treat the infectious disease at hand.

On the other hand, health camps or tents cater to a broader spectrum of needs during pandemics. Beyond offering immediate medical interventions, they often become centres for testing, vaccination drives and public health education. In regions where access to established medical facilities is limited, either due to geographical constraints or infrastructural limitations, these health camps bridge the gap, ensuring that even the most remote populations are kept in the lurch. Furthermore, health camps serve as vital nodes for data collection during outbreaks. Through these camps, health authorities can glean real‐time insights into the spread of the disease, its patterns and its impact. This data becomes instrumental in shaping immediate response strategies and predicting future trajectories of the outbreak.

During the COVID‐19 pandemic, Australia's approach to field hospital placement and utilisation was part of its broader public health response, which included a range of measures from strict lockdowns to comprehensive testing and contact tracing efforts. The deployment of field hospitals was one aspect of preparing the healthcare system to manage potential surges in COVID‐19 cases. In Melbourne, a field hospital was set up on the city's showgrounds to provide the curfewed residents with care if needed [20]. Similarly, in Canberra, the ACT government was opening a field hospital to prepare for an expected boom in cases of COVID‐19 [21]. Meanwhile, in Sydney, health authorities were preparing for a worst‐case scenario of COVID‐19 with a makeshift hospital at Sydney's Olympic Park [22]. The pandemic underscored the importance of having flexible and scalable healthcare solutions. Field hospitals could be quickly adapted to meet changing needs, which is crucial in the face of an unpredictable virus.

Internationally, China's rapid construction and deployment of field hospitals, known as Fangcang shelter hospitals, were critical to its response strategy. These facilities played a crucial role in managing the outbreak, especially in Wuhan, the initial epicentre of the pandemic. The locations of Fangcang Shelter Hospital are distinct from regular hospitals in terms of location requirements for admission and treatment of patients with epidemic diseases. Some factors must be considered in order to select the locations of the new Fangcang Shelter Hospital, such as the availability of large, open spaces that can accommodate a significant number of beds, the chosen location must be easily accessible, the necessary infrastructure, the potential impact on the surrounding community and ensuring compliance with local laws and regulations [15, 23, 24]. The Chinese experience with Fangcang Shelter hospitals offers valuable lessons for global pandemic preparedness [15]. The model demonstrated how rapid, scalable and flexible healthcare responses can be implemented in the face of an escalating public health crisis. The success of these hospitals in managing patient overflow, providing essential medical care and containing the virus's spread highlights their potential utility in future health emergencies. By providing immediate isolation for a large number of patients with mild‐to‐moderate symptoms, these hospitals played a critical role in breaking the chain of transmission. Additionally, Fangcang shelter hospitals served as triage centres, where patients could be assessed and either admitted for further care or referred to other healthcare facilities for more specialised treatment.

In the United Kingdom, the NHS responded by rapidly establishing seven temporary hospitals known as NHS Nightingale Hospitals [14]. NHS Nightingale Hospitals were primarily designed to provide care for COVID‐19 patients, particularly those who required intensive care. Similar to the Fangcang hospitals in China, a primary criterion for the NHS Nightingale Hospitals was the availability of large space [25]. These hospitals were equipped with the necessary medical infrastructure, including ventilators and oxygen supplies. The collaborative efforts included various stakeholders, including the military, private contractors and healthcare professionals [14]. The rapid development of these facilities showcased the agility of the NHS and its partners in responding to an emergency of unprecedented scale.

In the USA, the Commonwealth of Massachusetts undertook a significant healthcare initiative to bolster its medical infrastructure during the COVID‐19 pandemic [13]. In 9 days, a 500‐bed PAC facility was ready and innovatively located within the local convention centre. Five elements affected the selection process for the location: recommendations from healthcare professionals, the availability of healthcare services, the smooth transition and coordination between different levels of care, the preferences and past experiences of patients and their acquaintances and financial considerations [26]. This strategic decision directly responded to the escalating need for medical care facilities capable of handling the surge in COVID‐19 cases. By leveraging the expansive space of the convention centre, the response team addressed the urgent need for additional patient care capacity, particularly for those in the post‐acute phase of COVID‐19 treatment.

Establishing field hospitals in response to health emergencies encompasses a multifaceted array of challenges that necessitate comprehensive strategic planning and coordination. Logistical hurdles, including the rapid assembly of infrastructure and securing locations that support essential services, present significant operational complexities. Additionally, the endeavour faces considerable resource allocation challenges, competing for medical supplies, PPE and essential pharmaceuticals during times when global demand surges. Staffing these temporary facilities adequately with trained healthcare personnel and support staff, while avoiding burnout emerges as a critical barrier. Financial constraints further complicate the scenario, with substantial equipment, supplies and personnel investments potentially straining healthcare budgets. Regulatory and compliance issues, ensuring patient privacy and adherence to healthcare standards, demand vigilant oversight. Environmental and site‐specific considerations, such as weather conditions and accessibility, impact the functionality and efficiency of these temporary facilities. Addressing these barriers is critical to the successful deployment of field hospitals, underscoring the importance of integrated planning and collaboration among all stakeholders involved in healthcare emergency preparedness and response.

The study's recommendations are primarily based on the COVID‐19 pandemic experience. Future pandemics may have different characteristics, such as higher transmission rates or differing morbidity profiles, which could affect the adequacy of the proposed field hospital network. Aboriginal and Torres Strait Islander peoples have unique health needs and face higher rates of certain health conditions. The design and services offered by field hospitals must be tailored to address these specific health profiles, ensuring that medical care is relevant and effective in meeting the community's needs. The assumption that a 7.5 km radius is the most accessible distance for both pedestrians and motorists is based on previously published studies, which may not accurately reflect the current or future state of transportation infrastructure or travel behaviours in Australia. The study does not account for the availability of healthcare professionals and logistical support staff needed to operationalise and manage these field hospitals, which could limit the practical implementation of the recommendations.

## CONCLUSION

The comprehensive spatial analysis conducted in this study highlights the significant potential of strategically placed field hospitals in enhancing the preparedness and resilience of Australia's public health system against national or local emergencies. By proposing the establishment of 91 field hospitals across Australia's major capital city regions, the research suggests that over 95% of the urban population would be within a 7.5 km radius of an emergency healthcare facility. This extensive network of proposed sites, catering to varying population densities, ensures that both densely populated urban centres and less populated areas are adequately served. The study offers a blueprint for emergency healthcare deployment and significantly contributes to the discourse on public health logistics. The breadth of coverage provided by these temporary facilities outlines a robust strategy, poised to protect public health across the diverse demographic and geographic expanse of Australia's capital cities.

## AUTHOR CONTRIBUTIONS

Mazen Baazeem, Estie Kruger and Marc Tennant wrote the main manuscript text. Mazen Baazeem analysed the data and prepared the tables and figures. All authors reviewed the manuscript.

## CONFLICT OF INTEREST STATEMENT

The authors declare that they have no conflicts of interest.

## FUNDING INFORMATION

This research did not receive any specific grant from funding agencies in the public, commercial or not‐for‐profit sectors.

## ETHIC STATEMENT

Exemption from ethics review was obtained from the Human Research Ethics Committee at the University of Western Australia (approval number – 2021/ET000358).

## Data Availability

The data that support this study are available on the ABS website: https://www.abs.gov.au/census/find‐census‐data/datapacks.
